# Enhancing Chemical Stability and Bioavailability of Aneratrigine Capsules via Dry Granulation: Addressing Stability Challenges in Sodium Bicarbonate-Containing Formulations for Clinical Development

**DOI:** 10.3390/pharmaceutics17081047

**Published:** 2025-08-12

**Authors:** Kwan-Ik Cha, Ga-Eon Kim, Ji-Hyung Seol, Dong-Woo Kim, Seungbeom Lee

**Affiliations:** 1 New Drug Formulation Team, iN Therapeutics Co., Ltd., Yongin-si 17028, Republic of Korea; k.i.cha@intherapeutics.com (K.-I.C.); rkdjs1010@intherapeutics.com (G.-E.K.); jhseol10@intherapeutics.com (J.-H.S.); 2 Nonclinical Research Center, iN Therapeutics Co., Ltd., Yongin-si 17028, Republic of Korea; dwkim@intherapeutics.com; 3 Medicinal Chemistry and New Drug Formulation Center, iN Therapeutics Co., Ltd., Yongin-si 17028, Republic of Korea

**Keywords:** Nav1.7 inhibitor, aneratrigine, dry granulation, alkalizing agent, design of experiments

## Abstract

**Background**: Aneratrigine, a potent selective Nav1.7 inhibitor, faced challenges in developing a clinically viable oral formulation due to its poor aqueous solubility in acidic gastric conditions (0.06 mg/mL at pH 1.2), leading to limited bioavailability in Phase 1 studies. **Methods:** To address this, a capsule formulation containing sodium bicarbonate (NaHCO_3_) was developed to enhance dissolution via in situ pH modulation. However, production-scale wet granulation led to stability issues, such as capsule content discoloration and excessive degradant formation, attributed to NaHCO_3_ decomposition under thermal and moisture stress. This raised the content pH and triggered degradation products not seen in initial compatibility tests. Consequently, dry granulation was adopted to minimize heat and moisture exposure. **Results:** The dry granulation process proved scalable, maintaining chemical integrity across laboratory (1.5 kg), pilot (5.4 kg), and commercial (25.9 kg) batches. The optimized formulation showed enhanced stability (total impurities < 0.05%) and improved dissolution (>80% at 30 min, pH 4.0). **Conclusions:** This work establishes a robust manufacturing platform that overcomes stability challenges in alkalizer-containing formulations, facilitating the successful advancement of aneratrigine to Phase 2a and providing a model for developing heat- and moisture-sensitive compounds.

## 1. Introduction

Opioid analgesics, such as fentanyl, are widely used for managing moderate-to-severe acute pain but present significant safety risks and societal challenges despite their efficacy [1,2]. Fentanyl, for instance, is 50–100 times more potent than morphine but has a narrow therapeutic index (with an anticipated lethal dose of approximately 2 mg in humans) and a high potential for addiction [3,4,5,6]. In South Korea, long-term opioid users show a dependency rate of 21%, even though per capita opioid consumption (55 mg/year) is considerably lower than the OECD average (258 mg) and the U.S. average (678 mg) [7]. These data highlight the urgent need for non-opioid analgesics that lack addiction risks. The global market for non-opioid analgesics is projected to reach approximately USD 85 billion by 2030, underscoring the significant unmet medical demand [8].

In January 2025, the FDA approved suzetrigine (Journavx™, Vertex Pharmaceuticals), a selective Nav1.8 inhibitor, marking the first novel non-opioid treatment for acute pain in two decades. Its mechanism, which avoids central nervous system involvement, eliminates the risks of respiratory depression and addiction [9]. This milestone validated voltage-gated sodium channels (Nav) as viable targets for analgesics, spurring increased focus on Nav1.7, a critical regulator of peripheral nociceptors [10,11,12]. Genetic studies have shown that loss-of-function mutations in Nav1.7 lead to pain insensitivity, whereas gain-of-function mutations result in severe pain syndromes, providing robust genetic evidence for Nav1.7 as a therapeutic target for pain relief [13,14,15].

Aneratrigine, a potent and selective Nav1.7 inhibitor developed by our group (IC_50_ = 19 nM against Nav1.7, comparable to PF-05989771 [IC_50_ = 11 nM]), emerged as a promising candidate for an opioid alternative. The aneratrigine mesylate used in the investigational medicinal product (clinical formulation) is classified as a BCS class IV compound, characterized by low solubility and low permeability. This classification is based on its minimal solubility of 0.03 mg/mL at gastric pH 1.2, the pH at which solubility is lowest. In aqueous media, aneratrigine mesylate exhibits a solubility of 4.91 mg/mL, but its solubility is strongly pH-dependent due to multiple dissociation constants. According to ACD/Percepta simulations (v14.53.0), it has four pKa values: pK_a1_ = 1.1, pK_a2_ = 3.0, acidic pK_a3_ = 5.5, and basic pK_a4_ = 8.7, resulting in markedly variable solubility across different pH conditions. For example, solubility increases to a maximum of 5.22 mg/mL at pH 4.0, resembling the slightly acidic environment of the upper duodenum (a major absorption site), but decreases again to 0.71 mg/mL at intestinal pH 6.8. Furthermore, aneratrigine mesylate is highly hydrophilic, with a partition coefficient (log P) of −0.54 (in water, 37 °C) and a distribution coefficient (log D) ranging from −0.76 to 0.83 across pH 1 to 12, reflecting its limited membrane permeability. These physicochemical properties emphasize that formulation strategies for aneratrigine mesylate must carefully accommodate its low solubility at gastric pH and its pH-dependent dissolution behavior to optimize oral bioavailability.

To address this, initial formulation efforts incorporated 20% sodium bicarbonate (NaHCO_3_) via wet granulation, which improved dissolution (82.6% release at 30 min vs. 38.7% without an alkalizing agent) by neutralizing gastric pH. However, scaling up to production batches (25.9 kg) introduced critical stability issues, including the discoloration of capsule contents (from white to pale yellow) and impurities exceeding the specified limit. At the 1-month accelerated stability test, several impurities surpassed their individual threshold of 0.2%—with relative retention times (RRTs) and amounts as follows: 0.92 (0.38%), 2.40 (0.32%), and 2.44 (0.27%). The total impurity level reached 1.3%, exceeding the overall specification limit of 1.0%. These issues were attributed to the decomposition of NaHCO_3_ during prolonged drying (>16 h at 60 °C), which generated NaOH and catalyzed degradation pathways. In contrast, laboratory-scale batches (0.72 kg) with shorter drying times (5 h) exhibited no such instability, confirming that the formulation is sensitive to thermal and moisture stress at larger scales.

Recent advances in pharmaceutical formulation have provided several alternative approaches that replace traditional manufacturing methods such as wet granulation, addressing the poor aqueous solubility and stability of drug substances. Common strategies include the use of solid dispersions, where the drug is molecularly dispersed within a polymeric carrier matrix to enhance dissolution; lipid-based formulations, such as self-micro emulsifying drug delivery systems (SMEDDS), which improve the solubilization and absorption of lipophilic drugs; and amorphous formulations that eliminate crystallinity to increase apparent solubility. While these advanced technologies can effectively improve the bioavailability of poorly soluble drugs, they often involve additional regulatory and manufacturing complexities. In this study, we selected dry granulation as a robust and scalable approach requiring minimal changes to the existing composition. This allowed us to balance formulation performance with an efficient development and regulatory pathway.

For these reasons, this study implemented dry granulation, a process that minimizes heat and moisture exposure, thereby enhancing solubility while stabilizing the NaHCO_3_-containing formulation. This approach was successfully demonstrated across laboratory (1.5 kg), pilot (5.4 kg), and production (25.9 kg) scales, establishing a robust manufacturing platform for Aneratrigine’s advancement to Phase 2a trials.

## 2. Materials and Methods

### 2.1. Materials

Aneratrigine mesylate, the active pharmaceutical ingredient (API), was synthesized and supplied by an external contract manufacturing organization. The excipients used included spray-dried lactose (Fast Flo^®^ 316, Kerry Group, Tralee, Ireland), granulated lactose (Supertab^®^ 30GR, DFE Pharma, Nörten-Hardenberg, Germany), microcrystalline cellulose PH102 (Avicel^®^ PH102, Merck, Westpoint, PA, USA), sodium bicarbonate (Emprove^®^ Essential, Merck), croscarmellose sodium (Ac-di-sol^®^ SD-711, Dupont), magnesium stearate (Ligamed^®^ MF-2V-MB, Peter Greven GmbH, Venlo, Netherlands), and colloidal silicon dioxide (Aerosil^®^ 200 Pharma, Evonik Industries, Rheinfelden, Germany). Size 00 white gelatin capsules were sourced from Capsugel (Greenwood, SC, USA). All reagents and solvents were of HPLC grade, with acetonitrile and methanol obtained from Honeywell (Muskegon, MI, USA), and acetic acid, phosphoric acid, and sodium acetate from Samchun Chemical(Pyeongtaek, South Korea).

### 2.2. Laboratory-Scale Compatibility and Stress Testing

To investigate the root cause of impurity generation observed during large-scale wet granulation, a laboratory-scale accelerated stress test was designed to mimic the prolonged drying conditions of commercial-scale production. Table 1 summarizes the compositions of the reference capsule formulation (C1) and five experimental variants (C2–C6), each excluding a single excipient as a variable.

For each batch, the respective components were weighed and thoroughly mixed in a 100 mL beaker using a spatula. An excess amount of purified water (up to 10 mL per batch) was added to the mixture to simulate the large-scale wet granulation process, ensuring complete wetting. The beaker was sealed with parafilm to prevent water evaporation and placed in a drying chamber at 60 °C for 24 h to induce prolonged heat and moisture exposure. The dried granules were then passed through a #30 sieve (600 μm) and blended with extra-granular components as required. The final samples were sealed in 20 mL glass vials and stored under two conditions: accelerated (40 °C/75% relative humidity) and stress (60 °C, dry). Samples were withdrawn at 0, 2, and 4 weeks and analyzed for major impurities using the HPLC method described in Section 2.7 Impurity Analysis.

### 2.3. Preparation of Lab-Scale Dry Granules and Ejection Force Evaluation (Slugging Method)

In both the screening and formulation studies, approximately 14 g of sample—corresponding to 30 capsules per batch—was manufactured for each formulation (batch) as the experimental unit. The API and all excipients, except for magnesium stearate, were blended by bag mixing for 200 cycles. Magnesium stearate was finely sieved through a #30 mesh, weighed, and added to the pre-mixed powder, followed by an additional 100 cycles of bag mixing. The blended powder was then compressed using a single-punch tablet press (NP-RD10, Natoli Engineering Company, Inc., St Charles, MO, USA) equipped with a rectangular punch and die set (20 mm × 5 mm) at a compression force of 1.5 tons to produce slugs. The compression force applied during tableting was measured using a built-in hydraulic gauge. After compaction, the ejection force—that is, the effort required to remove the compacted slug from the die cavity via the lower punch—was assessed. Because the tablet press is manually operated, ejection performance was evaluated qualitatively based on the resistance felt by the operator. The following scale was used to describe the ejection force: “–” = No noticeable resistance during ejection (easily ejected), “+” = Slight force required, “++” = Noticeable resistance; ejection is somewhat difficult, “+++” = Ejection could not be completed even with considerable manual force. This qualitative ejection force assessment was used to compare the practical manufacturability of each slug formulation and to aid in optimizing excipient selection to prevent compaction or sticking issues during scale-up. After that, the slugs were coarsely milled and sieved through a #30 mesh to obtain granules of the desired size. Extra-granular components were then added to the granules and mixed by bag mixing to produce the final blend. Manual capsule filling was performed using a ProFiller 1100 (Torpac Inc., Fairfield, NJ, USA), loading the blend into size 00 white gelatin capsules.

### 2.4. Preparation of Production-Scale Dry Granules (Roller Compaction)

For production-scale dry granulation, all intra-granular components except magnesium stearate were loaded into a 60 L bin blender and pre-mixed at 20 ± 2 rpm for 10 min. The blended powder was then sieved through an automatics sieving machine equipped with a 0.800 mm screen (#24) at 1000 ± 100 rpm, followed by a secondary blending in the bin blender at 20 ± 2 rpm for 10 min. Magnesium stearate was sieved through a 0.630 mm mesh (#30), added to the bin blender, and mixed at 20 ± 2 rpm for 5 min for lubrication. The mixture was granulated using a WP120 roller compactor (Alexanderwerk AG und Alexanderwerk GmbH, Remscheid, Germany) equipped with knurled rolls, operating at a roller gap of 1.5 mm and a roller speed of 10 ± 1 rpm. The compaction force was set to 10 kN/cm^2^. The ribbons produced by roller compaction were then milled using the machine’s integrated two-stage rotor fine granulators in Diagonal-Design^®^. The milling process included both pre-granulation and fine-granulation stages, and the ribbons were passed through a 0.630 mm sieve (#30) at a rotor speed of 50 rpm. After granulation, the yield of dry granules was determined, and the required amounts of extra-granular components were recalculated and weighed accordingly. These were blended in the 60 L bin blender at 20 ± 2 rpm for 5 min. The granule and powder fractions were then homogenized in the same blender for 12 min after sieving through a 0.800 mm screen at 1000 ± 100 rpm using an automatic sieving machine. Final lubrication was performed by sieving magnesium stearate through a 0.630 mm mesh and blending at 20 ± 2 rpm for 5 min. Capsule filling was carried out using an automatic capsule filler (GKF 702, Bosch, Gerlingen, Germany) with the following parameters: dosing disk diameter 14.2 mm, machine speed 120 cycles/min, tamping pin height 10–12 mm, and tamping spring length 2 mm.

### 2.5. Evaluation of Slugs

#### 2.5.1. Tensile Strength of Slugs

The hardness of the slugs was measured using a Pharmatron Model 6D Tablet Tester (Dr. Schleuniger, Thun, Switzerland). The tensile strength was calculated based on the measured area and thickness of the slugs, and was used as an indicator of the compressibility of the blend.

(1) $$\sigma \left(Tensile\;strength\right)\left(MPa\right)=\frac{Crushing\;force\left(N\right)}{Cross-sectional\;Area\left({mm}^{2}\right)}$$

(2) $$=\frac{1}{9.807}(\frac{N}{kp})\times \frac{Crushing\;force(kp)}{Slug\;thickenss\left(mm\right)\times Slug\;width(mm)}$$

#### 2.5.2. Disintegration Testing of Slugs

Disintegration testing was conducted in accordance with USP <701> “Disintegration of Uncoated Tablets.” The slugs were placed in each tube of a standard disintegration apparatus (KDIT-200, Kukje engineering Co., Ltd. Paju, South Korea) The medium used was purified water maintained at 37 ± 2 °C. The apparatus was operated so that the basket-rack assembly moved up and down at a constant frequency (30–32 cycles per minute). The time at which no residue of the sample remained on the screen was recorded as the disintegration time.

### 2.6. Dissolution Testing of Capsules

The dissolution testing of capsules was performed using a USP Apparatus II (paddle method, 708-DS, Agilent Technologies, Inc., Craven Arms, United Kingdom) with 900 mL of pH 4.0 acetate buffer at 50 rpm and 37 ± 0.5 °C. Sinkers were used during dissolution testing. At predetermined time points (5, 10, 15, 30, 45, 60, 90, and 120 min), 5 mL samples were withdrawn and filtered through a 0.45 μm RC membrane filter (Sartorius, Göttingen, Germany). For the preparation of the standard solution, 27 mg of aneratrigine mesylate reference standard was accurately weighed, dissolved in 5 mL methanol, sonicated for 10 min, diluted to 100 mL, and filtered through a 0.45 μm RC membrane filter. HPLC analysis was performed using an Agilent 1260 Infinity II system with an Inertsil ODS 3V column (250 × 4.6 mm, 5 μm) or equivalent, using a mobile phase of 0.1% phosphoric acid in water and acetonitrile (70:30, *v*/*v*) at a flow rate of 1.0 mL/min. The detection wavelength was set at 287 nm, the injection volume was 2 μL, the column temperature was 25 °C, and the sample temperature was 5 °C. All dissolution values were corrected for the 5 mL sample taken at each time point, according to the following equations:

(3) $$C_{n}\left(\%\right)=\frac{A_{T}}{A_{S}}\times V_{0}\times C_{s}\times \frac{P}{L}$$

(4) $$D_{n}\left(mg\right)=V_{o}\times C_{n}+V_{s}\times \sum_{i=1}^{n-1}C_{i}$$

(5) $${\%\;Dissolved}_{n}\left(\%\right)=\frac{D_{n}}{L}\times 100\;$$

where $A_{T}$ is the peak area of the test solution, $A_{S}$ is the peak area of the standard solution, $V_{o}$ is the dissolution medium volume (900 mL), $C_{s}$ is the concentration of aneratrigine mesylate in the standard solution (mg/mL), *L* is the labeled content (mg/capsule), and *P* is the purity (%) of the aneratrigine mesylate reference standard. $C_{n}$ is the measured concentration of the drug in the dissolution vessel at sampling time point *n*. $D_{n}$ is the cumulative amount of drug dissolved at time point *n*, calculated by summing the drug present in the remaining medium and the total drug removed in all previous samples. $V_{s}$ is the sampling volume (5 mL).

### 2.7. Impurity Analysis

For impurity analysis, one capsule equivalent was accurately weighed and suspended in 500 mL of water:acetonitrile (75:25, *v*/*v*), stirred for at least 1 h, and filtered through a 0.45 μm RC membrane filter. The standard stock solution was prepared by dissolving 48 mg of aneratrigine mesylate reference standard in 100 mL of water:acetonitrile (75:25, *v*/*v*). Serial dilutions were made to obtain the working standard solution, which was also filtered through a 0.45 μm RC membrane filter. HPLC analysis(Arc™ HPLC, Waters, Milford, MA, USA) was performed under the following conditions: UV detection at 210 nm, Inertsil ODS 3V column (250 × 4.6 mm, 5 μm) or equivalent, mobile phase A (0.05% phosphoric acid in water), mobile phase B (acetonitrile), column temperature 25 °C, sample temperature 5 °C, injection volume 5 μL, and flow rate 1.0 mL/min. Integration was performed using Empower^®^ Pro software(version: 3.7.0). For individual impurities, they are reported only if their levels exceeded the reporting threshold of 0.05%, in accordance with regulatory guidance.

### 2.8. Statistical Analysis

Experimental design and statistical analysis were performed using Minitab^®^ 22 Statistical Software(Minitab^®^ 22.2.2). A full factorial design (2^3^ + 3 center points, total 11 runs) was employed to study the effects of formulation variables on slug tensile strength and dissolution rate at specific time points in pH 4.0 buffer. Based on the results, response optimization analysis was conducted to select the final formulation.

## 3. Results

### 3.1. Root-Cause Analysis of the Wet Granulation Process

To determine the cause of impurity formation observed during production-scale wet granulation, samples were subjected to prolonged heat and moisture exposure and then stored under accelerated (40 °C/75% RH) and stress (60 °C, dry) conditions. Visual inspection for discoloration and quantitative impurity analysis was conducted at the initial, 2-week, and 4-week time points.

#### 3.1.1. Discoloration

**Figure 1:**
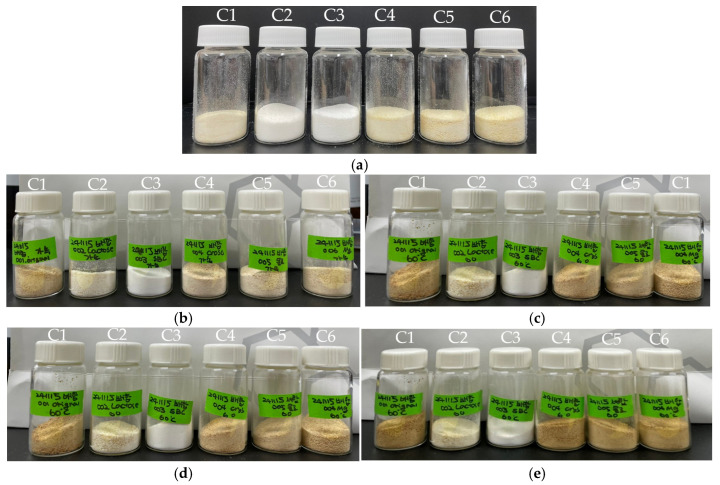
Discoloration of compatibility test samples: (**a**) Initial, (**b**) 2 weeks at 40 °C/75% RH, (**c**) 2 weeks at 60 °C, (**d**) 4 weeks at 40 °C/75% RH, (**e**) 4 weeks at 60 °C.

As shown in Figure 1, visual assessment revealed that all formulations except C3, which did not contain sodium bicarbonate, exhibited pronounced discoloration from the initial time point. In particular, formulations C1, C4, C5, and C6 displayed similarly severe discoloration, suggesting that interactions among the API, sodium bicarbonate, and lactose were responsible for this phenomenon.

#### 3.1.2. Impurities

Table 2 presents the individual impurities and the total impurity content for each sample (C1–C6) determined through impurity analysis under each condition. Regarding impurity levels, C3 (without NaHCO_3_) maintained total impurities below 0.2% under both storage conditions across all time points, showing a significant difference compared to the other formulations. In contrast, formulations containing NaHCO_3_ (C1, C2, C4–C6) exhibited a marked increase in total impurities, reaching 2.17–3.42% after four weeks at 60 °C.

### 3.2. Dry Granulation Formulation Study

The dry granulation formulation study was conducted at the laboratory scale using a single-punch tablet press (slugging method). For each formulation, the degree of granule formation was evaluated by measuring slug hardness, thickness, slug disintegration time, fines generation, and ejection pressure. The resulting granules were then blended according to the formulation and filled into capsules for dissolution testing.

#### Screening Experiments

**Figure 2:**
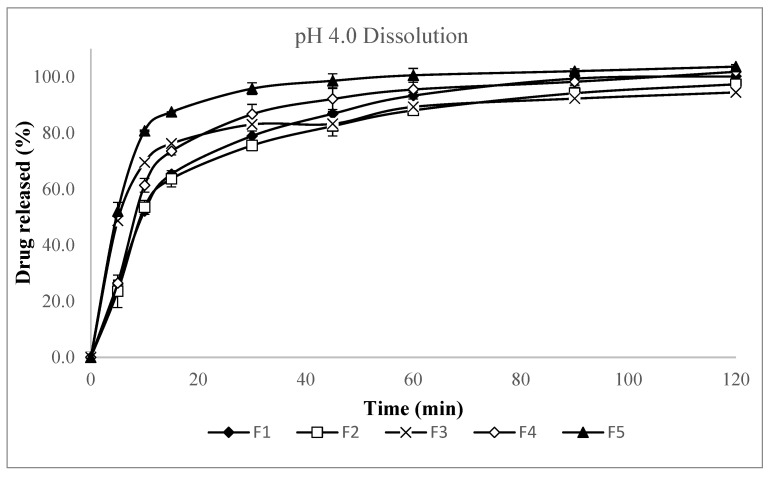
Dissolution profiles in pH 4.0 buffer (Apparatus 2, 50 rpm) for screening formulations F1–F5.

Several formulation variables were screened to optimize the dry granulation process, including the grade of lactose (spray-dried versus granulated), the inclusion of microcrystalline cellulose (MCC) within the granules, the percentage of sodium bicarbonate in the granules, and the addition of croscarmellose sodium as an intra-granular disintegrant. To evaluate these variables’ effects, formulations based on the original wet granulation composition were manufactured using the slugging method, as detailed in Table 3. The evaluation results of the slugs are presented in Table 4 and the dissolution results of the capsules in pH 4.0 buffer are presented in Figure 2 and Table A1.

The F1 formulation, which included 1% magnesium stearate based on the original wet granulation formulation, failed to achieve 80% dissolution within 30 min and generated a significant amount of fines during slugging. The F2 formulation, in which the lactose grade was changed from spray-dried lactose (Fast Flo^®^ 316) to granulated lactose (Supertab^®^ 30GR), exhibited the lowest dissolution rate (74.8% at 30 min), the lowest slug hardness, and the highest fines generation, making it unsuitable for dry granulation and thus excluded from further study. The F3 formulation, which contained intragranular MCC, produced the fewest fines and demonstrated improved slug formation, as well as a higher dissolution rate compared to F1. Increasing the sodium bicarbonate content from 10% to 20% in F4 did not significantly affect slug formation or disintegration, but resulted in a slightly faster dissolution rate at 30 min (85.8%) compared to F1. However, considering potential compatibility issues with the API, the sodium bicarbonate content was maintained at 10% as in the original wet granulation formulation. The F5 formulation, which included 2.5% intragranular croscarmellose sodium, achieved the highest dissolution rate at 30 min among F1–F5. Similarly to MCC, the inclusion of croscarmellose sodium as a disintegrant within the granules maintained its disintegration effect, leading to faster dissolution. However, F5 exhibited a higher ejection pressure during slugging compared to F1–F4. Based on these results, intragranular magnesium stearate percentage, intragranular MCC percentage, and intragranular croscarmellose sodium percentage were selected as key variables for further formulation optimization, considering both granule formation and processability as well as rapid dissolution.

### 3.3. Formulation Optimization

Based on the screening study, a full factorial design (2^3^ + 3 center points, 11 runs) using Minitab^®^ 22 Statistical Software(Minitab^®^ 22.2.2) was employed to optimize the formulation. The experimental design and the range and levels of each variable are presented in Table 5 and Table 6 (the detailed composition is shown in Table 7). The primary quality attributes evaluated were the dissolution rate at 10 min and 30 min in pH 4.0 buffer and slug tensile strength.

**Figure 3:**
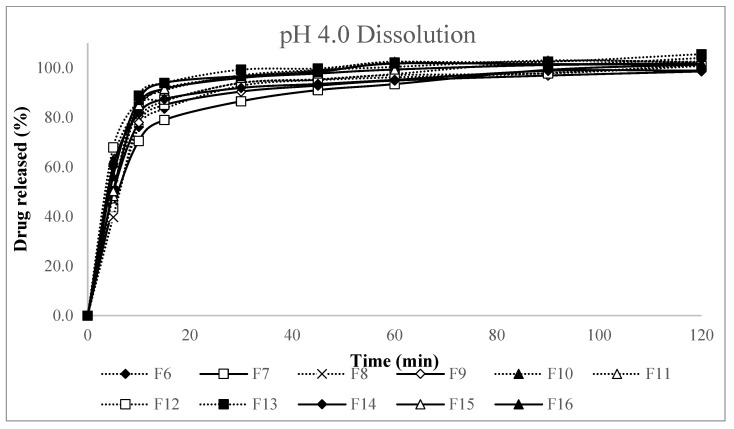
Dissolution profiles in pH 4.0 buffer for formulation study compositions F6–F16.

The formulation study for aneratrigine capsules using dry granulation evaluated the effects of three key variables—identified through screening studies—on dissolution in pH 4.0 buffer, slug disintegration, hardness, and ejection pressure. The slug evaluation results are presented in Table 8 and dissolution testing of capsules in pH 4.0 buffer was performed to evaluate the impact of each variable on drug release profiles (Table A2 and Figure 3).

To identify the primary factors influencing slug characteristics, regression analysis of slug tensile strength was performed through statistical modeling using Minitab^®^ 22 Statistical Software. Regression analysis of slug tensile strength indicated that intra-granular MCC percentage was the most significant factor influencing compressibility (*p* = 0.004) (Table A3), confirming that the MCC positively affected slug and tablet formation.

Based on dissolution test results, regression analysis via statistical modeling in Minitab^®^ 22 Statistical Software was conducted to identify key experimental factors influencing the formulation’s dissolution rate at 10 and 30 min in pH 4.0 buffer. Regression analysis demonstrated that intra-granular croscarmellose sodium was the most significant factor affecting dissolution at both 10 min (*p* = 0.004) (Table A4) and 30 min (*p* = 0.010) (Table A5). Increasing the amount of croscarmellose sodium improved the dissolution rate, but higher levels (as in F10–F13) resulted in excessive ejection pressure, making slug ejection difficult.

**Figure 4:**
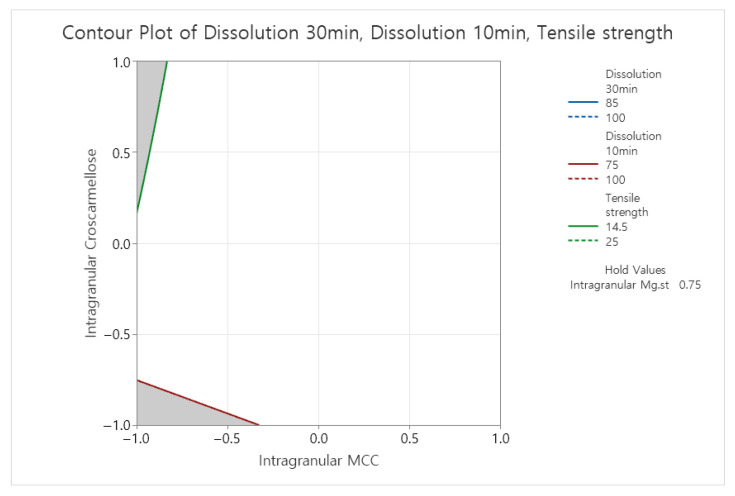
Contour plot analysis for final aneratrigine mesylate capsule composition. White region indicates acceptable tensile strength and dissolution (≥75% at 10 min, ≥85% at 30 min in pH 4.0 buffer and 14.5MPa≥ slug tensile strength).

To select the optimal formulation, contour plot analysis was performed using Minitab^®^ 22, with criteria set at ≥75% dissolution at 10 min, ≥85% at 30 min, and slug tensile strength ≥14.5 MPa (Figure 4).

**Figure 5:**
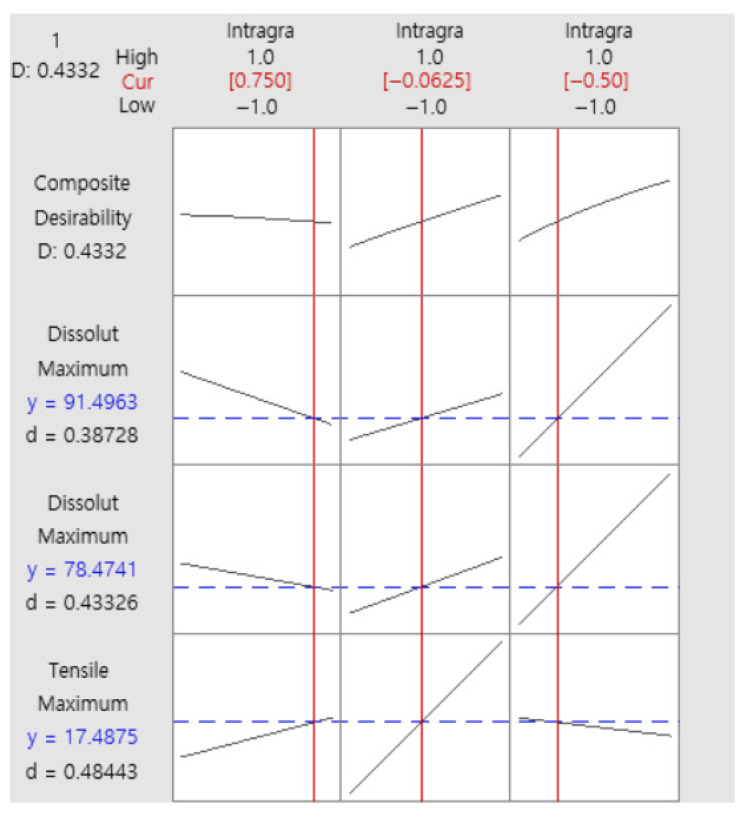
Response optimization analysis for final formulation. The red solid line represents the current setting values of each formulation factor within the range of −1 to +1. The black solid line indicates the trend of the response variable (such as tensile strength or dissolution rate) as the formulation factor changes. The blue solid line shows the expected response variable values based on the combination of the currently set formulation factors.

Based on statistical analysis and practical considerations such as ejection pressure, the final formulation was determined to contain 1.0% intra-granular magnesium stearate, 9.5% intra-granular MCC, and 2.5% intra-granular croscarmellose sodium (Table 9). Response optimization analysis for the formulation composition using Minitab^®^ 22 Statistical Software(Minitab^®^ 22.2.2) predicted a dissolution rate of 91.50% at 30 min in pH 4.0 buffer, meeting the target specification. The results of the response optimization analysis for the final formulation are presented in Figure 5.

### 3.4. Verification of Dry Granulation at Production Scale

The dry granulation process for the aneratrigine capsule formulation was successfully transferred to an external GMP-compliant manufacturing facility, where it was used to produce both a 5.4 kg non-GMP technical batch and a 25.9 kg GMP clinical batch. As summarized in Table 10, both production-scale batches met all internal quality specifications, including the dissolution profile (≥80% release within 30 min at pH 4.0), assay content, uniformity by mass variation (Acceptance value < 15), and microbial limits. Notably, the initial total impurity content was <0.05%, significantly below the acceptance criterion of 1.0%. These results confirm the robustness of the dry granulation manufacturing process and the stability of the active pharmaceutical ingredient (API) at production scale.

### 3.5. Stability Study Results

#### 3.5.1. Pilot-Scale Stability

At the pilot-scale batch (1.5 kg), the final formulation remained stable for six months under both long-term (25 °C/60% RH) (Table 11) and accelerated (40 °C/75% RH) (Table 12) conditions, with no evidence of discoloration, impurity increase (total impurities <0.05%), or delayed dissolution (≥80% dissolution at 30 min in pH 4.0 buffer, 50 rpm).

#### 3.5.2. Production-Scale Stability

For the 5.4 kg non-GMP batch, the formulation also maintained stability for three months under both long-term (Table 13) and accelerated conditions (Table 14), with no discoloration, impurity increase (total impurities <0.05%), or delayed dissolution observed.

## 4. Discussion

### 4.1. Decomposition Mechanism of Sodium Bicarbonate and Its Impact on Formulation Stability

The observed discoloration and elevated impurity levels in this study are directly attributable to the thermal decomposition of sodium bicarbonate. Upon exposure to moisture and temperatures exceeding 60 °C, NaHCO_3_ undergoes a two-stage decomposition reaction, resulting in the formation of NaOH [16].

(6) $$2NaHCO_{3}\to Na_{2}CO_{3}+H_{2}O+CO_{2}(Step1)\;Na_{2}CO_{3}+H_{2}O\to 2NaOH+CO_{2}\left(Step2\right)$$

The generation of NaOH elevates the pH within the formulation, which triggers Maillard reactions between the amine groups of the weakly basic drug and the carbonyl groups of reducing sugars, such as those present in lactose [17,18]. Notably, slight discoloration was observed in the C2 formulation, which lacks lactose, suggesting that reducing sugars in croscarmellose sodium may also contribute to these reactions. This indicates that formulations containing sodium bicarbonate require the rigorous evaluation of excipients that include reducing sugars (e.g., lactose, cellulose derivatives like HPMC) under conditions that simulate production-scale heat and moisture exposure.

### 4.2. Scientific Rationale for Process Change and Scalability

Transitioning from wet to dry granulation addresses a fundamental chemical instability issue rather than merely altering manufacturing methods. Sodium bicarbonate is indispensable for enhancing aneratrigine’s solubility in acidic gastric conditions and cannot be removed. Dry granulation minimizes heat/moisture exposure, effectively suppressing NaHCO_3_ decomposition—a well-established strategy for heat/moisture-sensitive compounds in pharmaceutical manufacturing.

### 4.3. Process-Dependent Functionality of Excipients

The critical difference between wet and dry granulation formulations lies in the incorporation of microcrystalline cellulose (MCC) within the granules. While MCC-containing formulations showed reduced dissolution in prior wet granulation studies (due to water-triggered loss of disintegrant functionality), they demonstrated accelerated dissolution in dry granulation. This reversal occurs because MCC retains its disintegrant properties when unexposed to water during processing [19,20]. Similarly, intra-granular croscarmellose sodium improved dissolution but increased ejection pressure during slugging. These findings demonstrate that excipient functionality is process-dependent, necessitating performance evaluations under actual manufacturing conditions.

### 4.4. Quality by Design (QbD) Optimization Strategy

The 2^3^ full factorial design with three center points provided an efficient framework for maximizing information with minimal experimental runs. Regression analysis confirmed MCC’s significant effect on slug tensile strength (*p* = 0.004) and croscarmellose sodium’s impact on dissolution (*p* = 0.004 at 10 min; *p* = 0.010 at 30 min). Contour plot analysis (Figure 4) enabled the identification of the optimal formulation space, aligning with QbD principles. This data-driven approach reduced empirical trial-and-error and established a scientific foundation for formulation development.

### 4.5. Successful Scale-Up and Technology Transfer

Consistent quality across scales (1.5 kg pilot to 5.4 kg non-GMP to 25.9 kg GMP batches) demonstrates the robustness of the dry granulation formulation. Total impurities remained <0.05% at all scales, confirming the effective mitigation of decomposition pathways. Successful technology transfer to an external GMP facility further validates the process’s versatility, ensuring manufacturing flexibility and supply chain reliability—critical factors for commercialization.

### 4.6. Stability Profile and Long-Term Quality Assurance

Stability studies across scales revealed no discoloration, impurity increases (<0.05% total impurities), or dissolution delays during the following:

- six-month accelerated/long-term testing [pilot-scale batch (1.5 kg)] (see Section 3.5.1)
- three-month accelerated/long-term testing [Technical (non-GMP) batch (5.4 kg)] (see Section 3.5.2)

This confirms that dry granulation fundamentally resolved the instability issues observed in wet granulation. Identical stability profiles at lab and production scales suggest predictable performance in future commercial manufacturing. This study exemplifies how process optimization can overcome stability challenges arising from excipient-API interactions. The dry granulation strategy provides a template for formulating heat/moisture-sensitive compounds, with implications extending beyond aneratrigine to similar alkaline-sensitive drug products.

## 5. Conclusions

This study successfully addressed a key challenge in developing an oral opioid alternative by improving the chemical stability of the aneratrigine capsule formulation, which incorporates sodium bicarbonate (NaHCO_3_) as a pH modifier. While the wet granulation process at production scale (25.9 kg) led to significant instability—manifested as discoloration and total impurities of up to 1.3% under accelerated conditions—the dry granulation process ensured excellent physicochemical stability across different scales. Specifically, laboratory-scale batches (1.5 kg) remained stable for 6 months, and production-scale batches (5.4 kg) for 3 months under both accelerated and long-term conditions, with no observed increase in impurities. These findings underscore the importance of minimizing heat and moisture exposure to prevent NaHCO_3_ decomposition and suggest that the wet granulation process may induce alkaline conditions conducive to amine-reducing sugar reactions, such as the Maillard reaction.

Having demonstrated excellent safety in Australian Phase 1 trials and secured Phase 2a approval in South Korea in 2024, aneratrigine stands out as a potent and selective Nav1.7 inhibitor. A key achievement of this work is the dry granulation formulation, which successfully overcame the poor solubility observed in acidic conditions during initial clinical studies. This formulation now ensures over 80% dissolution within 30 min at pH 4.0. The successful validation of process robustness at the GMP production scale further solidifies aneratrigine’s potential as a highly competitive next-generation non-opioid analgesic.

This development is particularly timely, aligning with the rising interest in Nav-targeted therapies, as evidenced by the FDA’s 2025 approval of suzetrigine (a Nav1.8 inhibitor). Our work directly contributes to meeting the critical societal need for non-addictive pain solutions. To advance aneratrigine’s global regulatory strategy, future work will involve dissolution-PK correlation studies in Phase 2b/3 trials and the collection of 36-month real-time stability data.

## Figures and Tables

**Table 1:** Composition of compatibility test formulations (C1–C6).

Component		Total Amount of Batch (g)					
		C1	C2	C3	C4	C5	C6
Intragranular	API (g)	4.79	4.79	4.79	4.79	4.79	4.79
	lactose monohydrate (g)	1.93	-	1.93	1.93	1.93	1.93
	sodium bicarbonate (g)	0.96	0.96	-	0.96	0.96	0.96
	DW (g)	q.s.					
Extragranular	sodium bicarbonate (g)	0.96	0.96	-	0.96	0.96	0.96
	lactose monohydrate (g)	0.29	0.00	0.29	0.29	0.29	0.29
	croscarmellose sodium (g)	0.48	0.48	0.48	-	0.48	0.48
	colloidal silicon dioxide (g)	0.10	0.10	0.10	0.10	-	0.10
	magnesium stearate (g)	0.10	0.10	0.10	0.10	0.10	-
Total		9.60	7.38	7.68	9.12	9.50	9.50

**Table 2:** Impurity analysis results from compatibility testing. Samples stored under accelerated (40 °C/75% RH) and stress (60 °C) conditions were analyzed by HPLC at 0, 2, and 4 weeks. Individual impurities are reported only if their levels exceeded the reporting threshold of 0.05%, in accordance with regulatory guidance.

Storage Condition/Time Point	RRT	Impurities (%)					
		C1	C2	C3	C4	C5	C6
Initial	0.49	0.29	0.05	-	0.25	0.17	0.14
	0.64	0.53	-	-	0.50	0.45	0.26
	1.13	0.08	-	-	0.06	-	-
	1.25	0.07	0.16	-	0.28	0.08	
	1.34	0.21	-	-	0.20	0.14	0.13
	2.49	-	0.05	-	-	-	-
	3.03	0.41	0.17	-	0.37	0.27	0.23
	3.08	0.26	0.13	-	0.24	0.19	0.16
	Total impurities	2.01	0.63	0.06	2.07	1.45	1.11
40 °C/75% RH/ 2 weeks	0.48	-	0.09	-	-	-	-
	0.51	0.34	0.09	-	0.28	0.19	0.12
	0.67	0.57	-	-	-	-	-
	0.90	0.22	-	-	0.15	0.13	0.09
	1.22	0.11	-	-	0.09	-	-
	1.31	0.12	-	-	-	0.08	
	2.24	0.06	0.06	-	0.06	-	-
	2.25	-	-	-	0.11	-	-
	2.24	-	-	-	0.08	-	-
	2.68	0.51	0.28	-	0.46	0.34	0.30
	Total impurities	2.01	0.53	0.00	1.74	1.20	0.76
60 °C/ 2 weeks	0.51	0.38	0.14	-	0.31	0.21	0.17
	0.56	0.10	0.15	-	-	-	-
	0.60	0.30	-	-	-	0.06	-
	0.67	0.40	-	-	0.41	0.38	0.21
	0.90	0.28	-	-	0.21	0.24	0.26
	1.22	0.08	-	-	0.06	-	-
	1.32	-	-	-		0.11	-
	2.17	-	-	-	0.06	-	-
	2.23	0.11	0.13	-	0.08	-	-
	2.39	-	-	-	0.11	-	-
	2.66	0.73	0.51	-	0.74	0.57	0.50
	Total impurities	2.44	0.97	0.00	2.01	1.57	1.14
40 °C/75% RH/ 4 weeks	0.48	0.06	0.09	-	-	-	-
	0.51	0.33	0.10	-	0.25	0.18	0.14
	0.67	0.49	-	-	0.42	0.40	0.23
	0.90	0.24	-	-	-	-	-
	1.21	0.16	-	-	0.16	-	0.12
	2.23	-	0.06	-		-	-
	2.40	0.08	0.08	-	0.06	-	-
	2.67	0.50	0.36	-	0.43	0.34	0.30
	2.72	0.37	0.27	-	0.31	0.26	0.21
	Total impurities	2.44	1.29	0.11	1.92	1.51	1.28
60 °C_/ 4 weeks	0.48	0.17	0.16	-	0.12	0.08	0.09
	0.51	0.44	0.18	-	0.31	0.20	0.18
	0.56	0.16	-	-	0.17	0.17	0.18
	0.67	0.16	-	-	0.23	0.18	0.11
	0.90	0.14	-	-	0.06	-	0.07
	0.96	-	0.06	-	-	-	-
	1.21	0.07	0.05	-	0.06	0.05	0.05
	1.45	0.09	-	-	-	-	-
	2.02	-	0.07	-	-	-	-
	2.23	0.06	0.11	-	0.06	-	-
	2.40	0.07	-	-	0.05	-	-
	2.67	0.90	0.61	-	0.77	0.59	0.54
	2.72	0.65	0.45	-	0.55	0.44	0.41
	Total impurities	3.42	2.17	0.10	2.85	2.25	2.11

**Table 3:** Formulations for screening studies. Variables modified from the original wet granulation composition: lactose grade (spray-dried vs. granulated), intragranular MCC inclusion, sodium bicarbonate percentage increase, and intra-granular croscarmellose sodium addition.

Component		Composition (mg/cps)				
		F1	F2	F3	F4	F5
Intragranular (Slug)	API	239.39	239.39	239.39	239.39	239.39
	lactose monohydrate (Fast Flo^®^)	96.51	-	48.51	96.51	96.51
	lactose monohydrate (Supertab^®^ 30GR)	-	96.51	-	-	-
	sodium bicarbonate	48.00	48.00	48.00	96.00	48.00
	microcrystalline cellulose	-	-	48.00	-	-
	croscarmellose sodium	-	-	-	-	12.00
	magnesium stearate	4.80	4.80	4.80	4.80	4.80
Extragranular	sodium bicarbonate	48.00	48.00	48.00	-	48.00
	lactose monohydrate (Fast Flo^®^)	14.50	-	14.50	14.50	14.50
	lactose monohydrate (Supertab^®^ 30GR)	-	14.50	-	-	-
	croscarmellose sodium	24.00	24.00	24.00	24.00	12.00
	colloidal silicon dioxide	4.80	4.80	4.80	4.80	4.80
	magnesium stearate	4.80	4.80	4.80	4.80	4.80
Total		484.80	484.80	484.80	484.80	484.80

**Table 4:** Slug evaluation results for each formulation. Slug weight, thickness, and hardness recorded; disintegration measured at 37 °C. Fines generation during ejection/surface: (–) negligible, (+) low, (++) moderate, (+++) high. Ejection force was qualitatively evaluated as follows: (–) no resistance, (+) slight resistance, (++) noticeable resistance, (+++) ejection very difficult or not possible by manual force. See Section 2.3 for details. All measurements were performed in triplicate.

Formulation no.	Slug Weight (mg)	Slug Thickness (mm)	Hardness (kp)	Tensile Strength (MPa)	Slug Disintegration Time	Fines Generation	Ejection Pressure
F1	824.0	3.21	20.39	12.46	>30 min (no change)	++	−
F2 (Supertab 30GR)	681.6	2.72	8.26	5.96	>30 min (no change)	+++	−
F3 (Intragranular MCC)	706.5	2.75	11.01	7.85	110 s	−	−
F4 (Intragranular Sodium bicarbonate −20%)	747.0	2.79	13.66	9.60	>30 min (no change)	+	−
F5 (Intragranular croscarmellose sodium)	663.3	2.59	13.76	10.42	30 s	+	+++

**Table 5:** Variable ranges and levels for full factorial design. Low level: −1, high level: +1, midpoint: 0.

Variables		Range	Level
1	intragranular magnesium stearate	0.25–1.25% (*w*/*w*)	−1, 0, +1
2	intragranular MCC	2–18% (*w*/*w*)	−1, 0, +1
3	intragranular croscarmellose sodium	1–5% (*w*/*w*)	−1, 0, +1

**Table 6:** Experimental design for formulation optimization. Run order randomized.

Formulation No.	Standard Order	Run Order	Center Point	Variable 1	Variable 2	Variable 3
F6	1	7	1	−1	−1	−1
F7	2	3	1	1	−1	−1
F8	3	1	1	−1	1	−1
F9	4	6	1	1	1	−1
F10	5	4	1	−1	−1	1
F11	6	11	1	1	−1	1
F12	7	8	1	−1	1	1
F13	8	5	1	1	1	1
F14	9	2	0	0	0	0
F15	10	9	0	0	0	0
F16	11	10	0	0	0	0

**Table 7:** Compositions tested in formulation study. Manufactured in randomized run order.

Component		Composition (mg/cps)										
		F6	F7	F8	F9	F10	F11	F12	F13	F14	F15	F16
Intragranular (Slug)	API	239.39	239.39	239.39	239.39	239.39	239.39	239.39	239.39	239.39	239.39	239.39
	Lactose Monohydrate, Fast Flo	86.91	86.91	10.11	10.11	86.91	86.91	10.11	10.11	48.51	48.51	48.51
	Sodium Hydrogen Carbonate, Emprove Essential	48.00	48.00	48.00	48.00	48.00	48.00	48.00	48.00	48.00	48.00	48.00
	Microcrystalline cellulose PH102	9.60	9.60	86.40	86.40	9.60	9.60	86.40	86.40	48.00	48.00	48.00
	Croscarmellose Sodium, Ac-di-sol SD-711	4.80	4.80	4.80	4.80	24.00	24.00	24.00	24.00	14.40	14.40	14.40
	Magnesium Stearate	1.20	6.00	1.20	6.00	1.20	6.00	1.20	6.00	3.60	3.60	3.60
Extragranular	Sodium Hydrogen Carbonate, Emprove Essential	48.00	48.00	48.00	48.00	48.00	48.00	48.00	48.00	48.00	48.00	48.00
	Lactose Monohydrate, Fast Flo	14.50	14.50	14.50	14.50	14.50	14.50	14.50	14.50	14.50	14.50	14.50
	Croscarmellose Sodium, Ac-di-sol SD-711	19.20	19.20	19.20	19.20	0.00	0.00	0.00	0.00	9.60	9.60	9.60
	Colloidal Anhydrous Silica, Aerosil 200 Pharma	4.80	4.80	4.80	4.80	4.80	4.80	4.80	4.80	4.80	4.80	4.80
	Magnesium Stearate	4.80	4.80	4.80	4.80	4.80	4.80	4.80	4.80	4.80	4.80	4.80

**Table 8:** Slug evaluation in the formulation study. Parameters and ejection pressure scale identical to Table 4 (n = 3). Fines generation was excluded from the evaluation criteria because it occurred rarely across all formulations (F6–F16).

Formulation no.	Slug Thickness (mm)	Hardness (kp)	Tensile Strength (MPa)	Disintegration Time (s)	Ejection Pressure
F6	2.75	21.6	15.4054	10	−
F7	2.64	20.6	15.3044	2	−
F8	2.27	20.7	17.8854	16	−
F9	2.50	28.2	22.1239	22	−
F10	2.36	15.8	13.1310	70	++
F11	2.41	18.4	14.9746	65	++
F12	2.18	22.4	20.1532	55	++
F13	2.18	22.3	20.0633	55	++
F14	2.55	20.6	15.8446	25	+
F15	2.57	20.7	15.7976	26	+
F16	2.54	20.5	15.8297	24	+

**Table 9:** Final composition for aneratrigine dry granulation capsules.

Function		Components	Composition (mg/cps)
Intragranular	API	aneratrigine mesylate	239.39 mg (equiv. to aneratrigine 200 mg as free base)
	Diluent	lactose monohydrate	48.31 mg
	Diluent	microcrystalline cellulose	45.80 mg
	Alkalizing agent	sodium bicarbonate	48.00 mg
	Disintegrant	croscarmellose sodium	12.00 mg
	Lubricant	magnesium stearate	4.80 mg
Extragranular	Diluent	lactose monohydrate	14.50 mg
	Alkalizing agent	sodium bicarbonate	48.00 mg
	Disintegrant	croscarmellose sodium	12.00 mg
	Glidant	colloidal silicon dioxide	4.80 mg
	Lubricant	magnesium stearate	2.40 mg
Total Capsule weight (mg)			484.80 mg

**Table 10:** Quality specifications and release data for production-scale aneratrigine capsules (non-GMP and GMP batches).

Test	Acceptance Criteria	Technical Batch, Non-GMP Batch	GMP Batch, For Clinical Trial
Appearance	White to off-white opaque capsules without deformation containing off-white or pale-yellow powder	Off-white opaque capsules without deformation containing white powder	Off-white opaque capsules without deformation containing white powder
Assay	95.0–105.0%	99.2%	97.3%
Related Substances	Individual Impurity NMT 0.2% Total Impurities NMT 1.0%	No impurities exceeded RL Total Impurities: <0.05%	No impurities exceeded RLTotal Impurities: <0.05%
Dissolution	Meets the requirement in USP <711>: NLT 80% (Q = 75) in 30 min	Minimum: 92% Maximum 97% Average: 94%	Minimum: 96% Maximum 98% Average: 97%
Uniformity of dosage units by mass variation	Meets the requirement in USP <905>: Report AV Value.	AV = 5.0	AV = 3.0
Microbial Limit	Total Aerobic Microbial Count (TAMC) ≤ 103 CFU/g	<100 CFU/g	<100 CFU/g
	Total Yeast and Mold Count (TYMC) ≤ 102 CFU/g	<100 CFU/g	<100 CFU/g
	*Escherichia coli* Absent/1g	Absent	Absent

**Table 11:** Long-term stability (25 °C/60% RH) of pilot-scale batch (6 months).

Test/Attribute	Acceptance Criteria	T = 0	T = 2W	T = 1M	T = 3M	T = 6M	T = 9M	T = 12M
Appearance	White to off-white opaque capsules	Off white opaque capsules	Off white opaque capsules	Off white opaque capsules	Off white opaque capsules	Off white opaque capsules	TBD	TBD
Assay	95.0–105.0%	102.8%	100.2%	99.3%	99.7%	100.3%	TBD	TBD
Impurities	Individual Impurity NMT 0.2%	N/D	N/D	N/D	N/D	N/D	TBD	TBD
	Total Impurities NMT 1.0%	N/D	N/D	N/D	0.14%	N/D	TBD	TBD
Dissolution	Meets the requirement in USP <711>: NLT 80% (Q = 75) in 30 min	Minimum: 90% Maximum: 99% Average: 93%	Minimum: 93% Maximum: 100% Average: 96%	Minimum: 90% Maximum: 95% Average: 92%	Minimum: 98% Maximum: 106% Average: 102%	Minimum: 87% Maximum: 98% Average: 93%	TBD	TBD
Uniformity of dosage units by mass variation	Meets the Requirement in USP <905>: Report AV Value	14.2 (L1)	N/T	N/T	N/T	N/T	N/T	N/T

**Table 12:** Accelerated stability (40 °C/75% RH) of pilot-scale batch (6 months).

Test/Attribute	Acceptance Criteria	T = 0	T = 2W	T = 1M	T = 3M	T = 6M
Appearance	White to off-white opaque capsules	Off white opaque capsules	Off white opaque capsules	Off white opaque capsules	Off white opaque capsules	Off white opaque capsules
Assay	95.0–105.0%	102.8%	101.3%	98.0%	99.8%	97.7%
Impurities	Individual Impurity NMT 0.2%	N/D	N/D	N/D	N/D	N/D
	Total Impurities NMT 1.0%	N/D	N/D	N/D	0.17%	N/D
Dissolution	Meets the requirement in USP <711>: NLT 80% (Q = 75) in 30 min	Minimum: 90% Maximum: 99% Average: 93%	Minimum: 86% Maximum: 93% Average: 90%	Minimum: 91% Maximum: 95% Average: 92%	Minimum: 91% Maximum: 102% Average: 95%	Minimum: 90% Maximum: 100% Average: 94%
Uniformity of dosage units by mass variation	Meets the Requirement in USP <905>: Report AV Value	14.2 (L1)	N/T	N.T	N.T	N.T

**Table 13:** Long-term stability (25 °C/60% RH) of production-scale batch (non-GMP, 5.4 kg; 3 months).

Test/Attribute	Acceptance Criteria	T = 0	T = 1M	T = 2M	T = 3M	T = 5M	T = 6M	T = 9M	T = 12M
Appearance	White to off-white opaque capsules containing off-white or pale-yellow powder	Off-white opaque capsules without deformation containing white powder	Off-white opaque capsules without deformation containing white powder	Off-white opaque capsules without deformation containing white powder	Off-white opaque capsules without deformation containing white powder	TBD	TBD	TBD	TBD
Assay	95.0–105.0%	99.2%	95.7%	96.4%	98.0%	TBD	TBD	TBD	TBD
Impurities	Individual Impurity NMT 0.2%	N/D	N/D	N/D	N/D	TBD	TBD	TBD	TBD
	Total Impurities NMT 1.0%	N/D	N/D	N/D	N/D	TBD	TBD	TBD	TBD
Dissolution	Meets the requirement in USP <711>: NLT 80% (Q = 75) in 30 min	Minimum: 92% Maximum: 97% Average: 94%	Minimum: 91% Maximum: 96% Average: 93%	Minimum: 89% Maximum: 100% Average: 97%	Minimum: 94% Maximum: 97% Average: 96%	TBD	TBD	TBD	TBD
Uniformity of dosage units by mass variation	Meets the Requirement in USP <905>: Report AV Value	AV= 5.0	N/T	N/T	N/T	N/T	N/T	N/T	N/T
Microbial Limit	Total Aerobic Microbial Count: NMT 10^3^ CFU/g	<100 CFU/g	N/T	N/T	N/T	N/T	TBD	N/T	TBD
	Total Yeast and Mold Count: NMT 10^2^ CFU/g	<100 CFU/g	N/T	N/T	N/T	N/T	TBD	N/T	TBD
	Escherichia coli: Absent	Complies	N/T	N/T	N/T	N/T	TBD	N/T	TBD

**Table 14:** Accelerated stability (40 °C/75% RH) of production-scale batch (non-GMP, 5.4 kg; 3 months).

Test/Attribute	Acceptance Criteria	T = 0	T = 1M	T = 2M	T = 3M	T = 5M	T = 6M
Appearance	White to off-white opaque capsules containing off-white or pale-yellow powder	Off-white opaque capsules without deformation containing white powder	Off-white opaque capsules without deformation containing white powder	Off-white opaque capsules without deformation containing white powder	Off-white opaque capsules without deformation containing white powder	TBD	TBD
Assay	95.0–105.0%	99.2%	95.5%	95.4%	97.2%	TBD	TBD
Impurities	Individual Impurity NMT 0.2%	N/D	N/D	N/D	N/D	TBD	TBD
	Total Impurities NMT 1.0%	N/D	N/D	N/D	N/D	TBD	TBD
Dissolution	Meets the requirement in USP <711>: NLT 80% (Q = 75) in 30 min	Minimum: 92% Maximum: 97% Average: 94%	Minimum: 87% Maximum: 99% Average: 95%	Minimum: 82% Maximum: 100% Average: 96%	Minimum: 93% Maximum: 98% Average: 96%	TBD	TBD
Uniformity of dosage units by mass variation	Meets the Requirement in USP <905>: Report AV Value	AV = 5.0	N/T	N/T	N/T	N/T	N/T
Microbial Limit	Total Aerobic Microbial Count: NMT 10^3^ CFU/g	<100 CFU/g	N/T	N/T	N/T	N/T	TBD
	Total Yeast and Mold Count: NMT 10^2^ CFU/g	<100 CFU/g	N/T	N/T	N/T	N/T	TBD
	Escherichia coli: Absent	Complies	N/T	N/T	N/T	N/T	TBD

**Figure A1:**
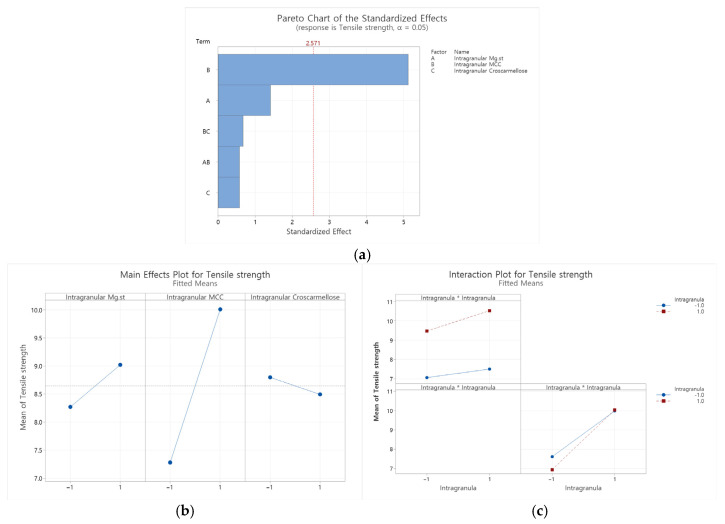
Statistical Analysis Results for Tensile Strength of Slugs: (**a**) Pareto Chart for Each Factor Affecting Tensile Strength of Slugs (α = 0.05); (**b**) Main Effects Plot for Each Factor Affecting Tensile Strength of Slugs; (**c**) Interaction Plot for Tensile Strength.

**Figure A2:**
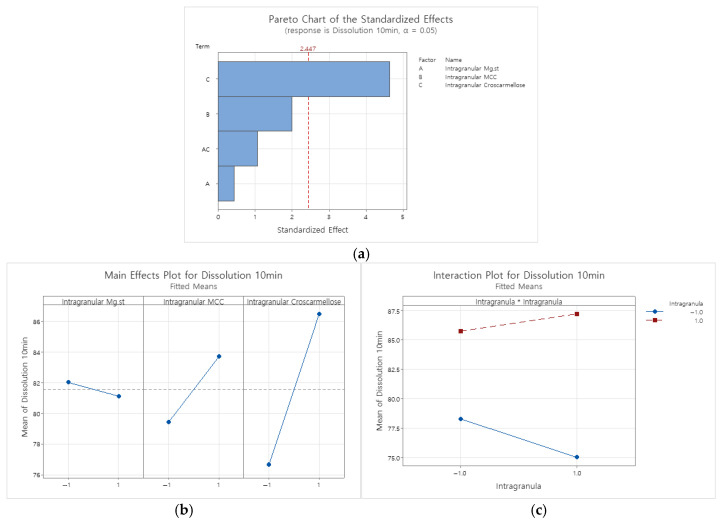
Statistical analysis results for the dissolution rate at 10 min in pH 4.0 buffer: (**a**) Pareto Chart of factors affecting the 10 min dissolution rate (α = 0.05); (**b**) Main Effects Plot of factors affecting the 10 min dissolution rate; (**c**) Interaction Plot for the 10 min dissolution rate in pH 4.0 buffer.

**Figure A3:**
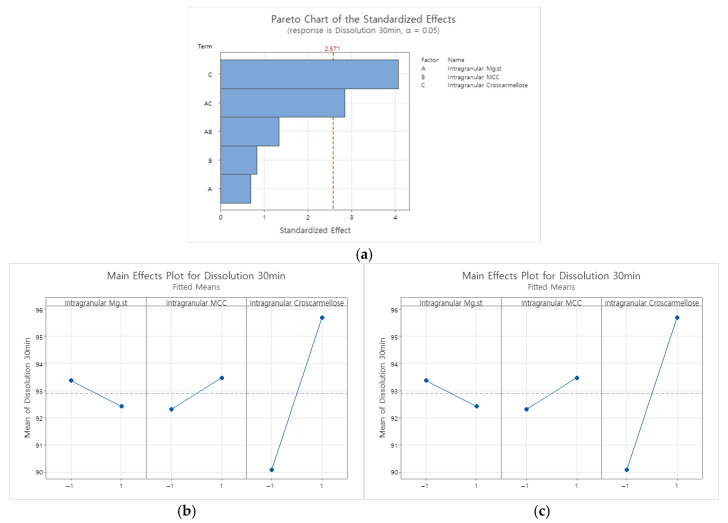
Statistical analysis results for dissolution rate at 30 min in pH 4.0 buffer: (**a**) Pareto Chart of factors affecting the 30 min dissolution rate (α = 0.05); (**b**) Main Effects Plot of factors affecting the 30 min dissolution rate; (**c**) Interaction Plot for the 30 min dissolution rate in pH 4.0 buffer.

## Data Availability

Data available on request from the authors.

## Abbreviations

The following abbreviations are used in this manuscript:

Nav	Voltage-Gated Sodium Channel
DoE	Design of Experiments
OECD	Organization for Economic Co-operation and Development
U.S	United States
FDA	Food and Drug
IC_50_	Half Maximal Inhibitory Concentration (the concentration of an inhibitor where the response is reduced by half)
pKa_1,2,3…_	Acid Dissociation Constant
API	Active pharmaceutical ingredient
DW	Distilled water
RH	Relative humidity
q.s	as much as needed
HPLC	High-Performance Liquid Chromatography
RC	Regenerated Cellulose
MCC	Microcrystalline Cellulose
AV	Acceptance value
RL	Report Limit
CFU	Colony Forming Unit
N/D	Not Detected
NMT	Not more than
N/T	Not Tested
TBD	To Be Determined

## Appendix A. pH 4.0 Dissolution Test Results

### Appendix A.1. pH 4.0 Dissolution Test Results: Screening Study (F1–F5), Formulation Study (F6–F16)

**Table A1:** Dissolution rates in pH 4.0 buffer (Apparatus 2, 50 rpm) for screening formulations F1–F5 (n = 3).

pH 4.0	Drug Release (%)							
Formulation No/Time	5 min	10 min	15 min	30 min	45 min	60 min	90 min	120 min
F1	26.2	52.2	65.3	78.9	86.9	93.3	99.4	100.2
F2	23.6	53.5	63.6	75.5	82.5	88.1	94.2	97.4
F3	48.7	69.5	76.2	83.0	83.3	89.3	92.3	94.5
F4	26.4	61.3	73.5	86.7	92.1	95.5	98.2	101.8
F5	52.1	80.8	87.5	95.8	98.6	100.6	102.0	103.7

**Table A2:** Dissolution rates in pH 4.0 buffer (Apparatus 2, 50 rpm) for formulations F6–F16.

pH 4.0	Drug Release (%)							
Formulation No/Time	5 min	10 min	15 min	30 min	45 min	60 min	90 min	120 min
F6	39.8	76.0	82.5	91.7	93.6	95.4	99.9	99.3
F7	43.6	70.3	77.9	85.5	89.5	91.5	96.7	98.3
F8	39.8	78.5	85.4	92.8	93.5	94.0	95.2	98.0
F9	49.5	77.7	83.9	89.3	91.2	92.7	94.2	95.4
F10	63.0	83.5	90.5	95.2	97.1	100.1	98.3	100.5
F11	48.3	83.8	89.7	95.8	97.4	98.1	100.2	98.8
F12	68.0	85.9	85.8	92.7	93.6	94.8	94.9	97.4
F13	50.3	88.6	92.7	98.0	98.0	99.8	99.6	102.1
F14	60.3	81.4	86.2	90.7	91.8	92.9	96.1	95.6
F15	50.3	84.8	90.3	94.7	96.0	97.0	98.3	98.1
F16	56.3	86.8	92.6	95.4	96.6	99.4	98.6	98.4

### Appendix B.2. Statistical Analysis Results (Minitab® 22 Statistic Software): Formulation Study (F6–F16)

#### Appendix B.2.1. Tensile Strength of Slugs

**Table A3:** ANOVA Results for Tensile Strength of Slugs.

Source	DF	Adj SS	Adj MS	F-Value	*p*-Value
Model	5	16.6548	3.3310	5.87	0.037
Linear	3	16.2098	5.4033	9.52	0.016
Intragranular Mg.st	1	1.1277	1.1277	1.99	0.218
Intragranular MCC	1	14.8954	14.8954	26.25	0.004
Intragranular Croscarmellose	1	0.1867	0.1867	0.33	0.591
2-Way Interactions	2	0.4450	0.2225	0.39	0.695
Intragranular Mg.st*Intragranular MCC	1	0.1881	0.1881	0.33	0.590
Intragranular MCC*Intragranular Croscarmellose	1	0.2568	0.2568	0.45	0.531
Error	5	2.8372	0.5674		
Curvature	1	1.3735	1.3735	3.75	0.125
Lack-of-Fit	2	1.4634	0.7317	4878.83	0.000
Pure Error	2	0.0003	0.0001		
Total	10	19.4920			

#### Appendix B.2.2. Dissolution Rate at 10 Min in pH 4.0 Buffer

**Table A4:** ANOVA Results for dissolution rate at 10 min in pH 4.0 Buffer.

Source	DF	Adj SS	Adj MS	F-Value	*p*-Value
Model	4	245.020	61.255	6.72	0.021
Linear	3	234.450	78.150	8.57	0.014
Intragranular Mg.st	1	1.711	1.711	0.19	0.680
Intragranular MCC	1	36.683	36.683	4.02	0.092
Intragranular Croscarmellose	1	196.056	196.056	21.51	0.004
2-Way Interactions	1	10.570	10.570	1.16	0.323
Intragranular Mg.st*Intragranular Croscarmellose	1	10.570	10.570	1.16	0.323
Error	6	54.698	9.116		
Curvature	1	31.633	31.633	6.86	0.047
Lack-of-Fit	3	8.270	2.757	0.37	0.785
Pure Error	2	14.795	7.397		
Total	10	299.718			

#### Appendix B.2.3. Dissolution Rate at 30 Min in pH 4.0 Buffer

**Table A5:** ANOVA results for dissolution rate at 30 min in pH 4.0 buffer.

Source	DF	Adj SS	Adj MS	F-Value	*p*-Value
Model	5	108.992	21.798	5.60	0.041
Linear	3	71.697	23.899	6.14	0.039
Intragranular Mg.st	1	1.944	1.944	0.50	0.511
Intragranular MCC	1	2.938	2.938	0.75	0.425
Intragranular Croscarmellose	1	66.815	66.815	17.17	0.009
2-Way Interactions	2	37.296	18.648	4.79	0.069
Intragranular Mg.st*Intragranular MCC	1	7.095	7.095	1.82	0.235
Intragranular Mg.st*Intragranular Croscarmellose	1	30.201	30.201	7.76	0.039
Error	5	19.461	3.892		
Curvature	1	2.390	2.390	0.56	0.496
Lack-of-Fit	2	3.890	1.945	0.30	0.772
Pure Error	2	13.181	6.590		
Total	10	128.453			
